# ReseqChip: Automated integration of multiple local context probe data from the MitoChip array in mitochondrial DNA sequence assembly

**DOI:** 10.1186/1471-2105-10-440

**Published:** 2009-12-22

**Authors:** Marian Thieme, Claudio Lottaz, Harald Niederstätter, Walther Parson, Rainer Spang, Peter J Oefner

**Affiliations:** 1 Institute of Functional Genomics, University of Regensburg, Josef-Engert-Str. 9, D-93053 Regensburg, Germany; 2 Institute of Legal Medicine, Innsbruck Medical University, Muellerstrasse 44, A-6020 Innsbruck, Austria

## Abstract

**Background:**

The Affymetrix MitoChip v2.0 is an oligonucleotide tiling array for the resequencing of the human mitochondrial (mt) genome. For each of 16,569 nucleotide positions of the mt genome it holds two sets of four 25-mer probes each that match the heavy and the light strand of a reference mt genome and vary only at their central position to interrogate all four possible alleles. In addition, the MitoChip v2.0 carries alternative local context probes to account for known mtDNA variants. These probes have been neglected in most studies due to the lack of software for their automated analysis.

**Results:**

We provide ReseqChip, a free software that automates the process of resequencing mtDNA using multiple local context probes on the MitoChip v2.0. ReseqChip significantly improves base call rate and sequence accuracy. ReseqChip is available at http://code.open-bio.org/svnweb/index.cgi/bioperl/browse/bioperl-live/trunk/Bio/Microarray/Tools/.

**Conclusions:**

ReseqChip allows for the automated consolidation of base calls from alternative local mt genome context probes. It thereby improves the accuracy of resequencing, while reducing the number of non-called bases.

## Background

The human mitochondrial (mt) DNA is a double-stranded circular molecule of 16,569 base pairs (bp) and consists of two parts: The non-coding displacement loop, also referred to as the control region, and the coding region. The control region is 1,124 bp in size and encompasses the nucleotide positions (nps) 16,024 to 576. It contains transcription and replication elements. The hypervariable segments HVS I (nps 16,024-16,383) and HVS II (nps 57-372) within the control region are hotspots for mtDNA alterations. The mutation rate of the hypervariable segments is tenfold higher than that of the coding region [1], whose mutation rate is already 10 times higher than that of nuclear genomic DNA because of the lack of protective histones, inefficient DNA repair systems and continuous exposure to mutagenic effects of oxygen radicals generated by oxidative phosphorylation [2]. The mtDNA coding region, on the other hand, contains 37 genes coding for two ribosomal RNAs and 22 transfer RNAs, which are required for intramitochondrial translation, as well as 13 polypeptides, which are components of the respiratory chain enzyme complexes in the inner membrane of the mitochondria that are essential in the energy production of the human cell. While most human cells contain two copies of nuclear DNA, they may possess up to 100,000 copies of mtDNA [3]. The majority of these copies are identical or homoplasmic immediately after birth. Somatic mutations will give rise to variant mtDNA copies or heteroplasmy over a lifetime, possibly contributing, for example, to the initiation and progression of cancer [4]. Mutant copies may also be inherited and are known to cause a variety of diseases affecting mostly the energy-hungry cells of the muscles, brain, or nerves [5]. The large number of copies and polymorphisms has made mtDNA particularly useful in forensics [6] and paleogenetics [7] where the amount of nuclear DNA in a sample is limited or degraded. Finally, due to its maternal inheritance and the lack of recombination, mtDNA provides insights into the maternal history of anatomically modern humans and into the impact of genetic drift, demography and selection on the level, kind and distribution of polymorphism observed in extant mtDNA lineages [8]. The analysis of mtDNA sequence variation is therefore of common interest in medical, forensic and population genetics. Re-sequencing of mtDNA has been traditionally accomplished by PCR amplification of the mitochondrial genome in approximately 40 overlapping fragments followed by uni- or preferentially bidirectional sequencing [8]. Although modern capillary array sequencers allow the analysis of as many as 2,300 sequencing reactions or an equivalent of 29 mitochondrial genomes per day, the complexity of conventional sequence analysis has spurred the development of novel tools such as the Affymetrix oligonucleotide-based array MitoChip for the detection of mtDNA variation. The first version of MitoChip was introduced in 2004. It was capable of sequencing the mtDNA coding region in a single hybridization after its long-range amplification in three overlapping fragments [9]. A more recent version (v2.0) of MitoChip covers in addition the control region. More importantly, it carries probes that match not only a reference mtDNA (the so-called revised Cambridge Reference Sequence (rCRS) [10]), but also common variants thereof, 99% of which are located in the hypervariable segments of the control region. The MitoChip v2.0 has been applied mainly to the detection of somatic mutations in cancer [11-17]. In addition, it has been used in forensic and population genetic studies [18,19].

**Figure 1:**
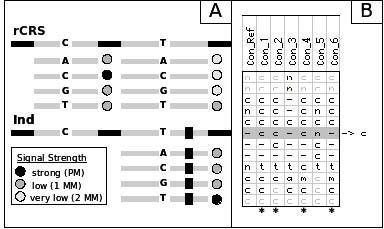
**Concept of multiple local context probes**. (**A**) Three probe sets for 3 different sequence contexts are shown for one strand only. If an individual's (Ind) genome contains a nucleotide position different from the reference (rCRS) within 12 nucleotide positions (nps) on either side of the interrogated position, only one of the alternative probes in the lower right corner will yield a strong hybridization signal. (**B**) Section of an alignment of additional fragments to reference sequence (left-most column). Due to the specified settings only fragments marked by a star fulfil the required reliability of no N-calls within 3 nps on either side of the interrogated position.

**Figure 2:**
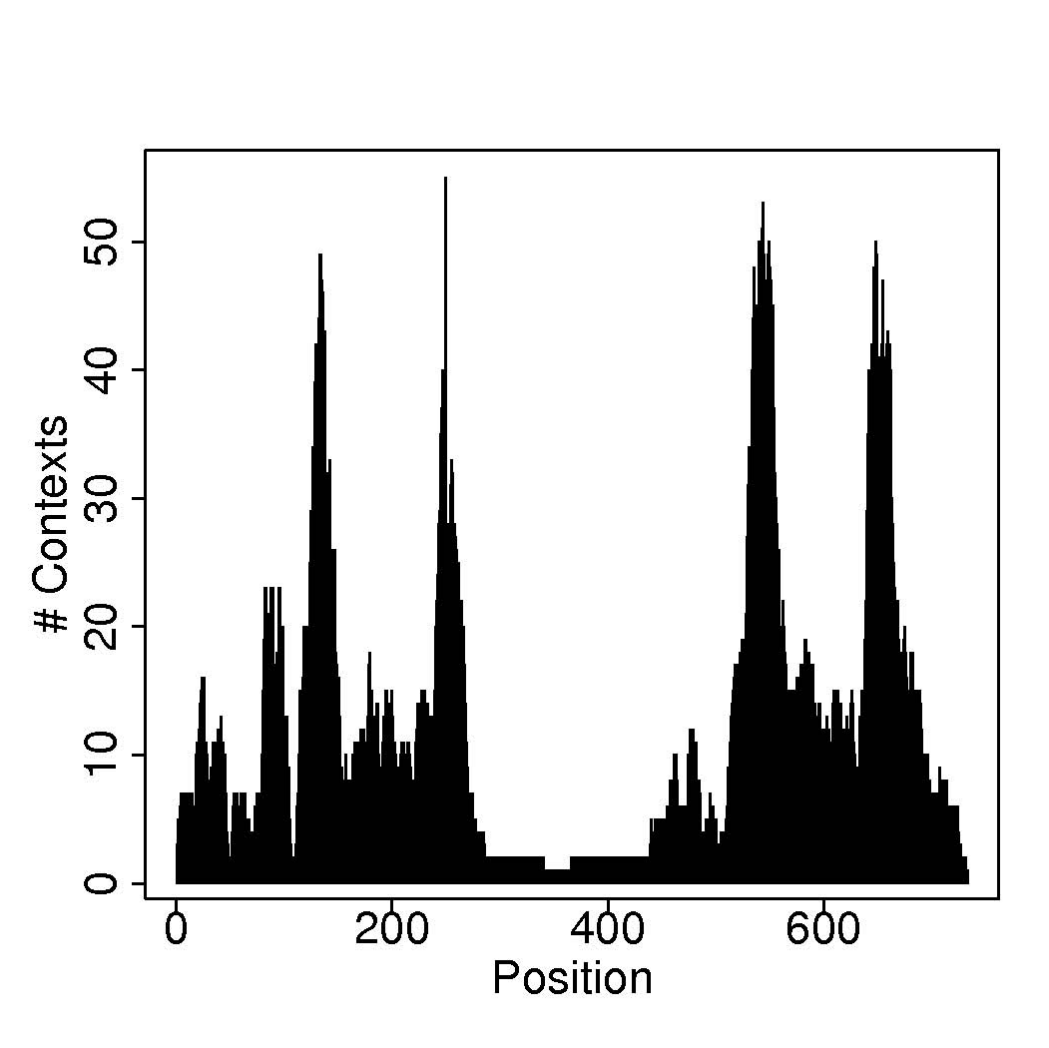
**Different Contexts**. Number of multiple contexts available by MitoChip v2.0 for the 733 nucleotide positions of the rCRS for which multiple local context probes are present. Note that the contexts of a certain position can vary at several of its 12 flanking bases up- and downstream of the center.

As illustrated in Figure 1A, microarray-based re-sequencing is done in a local sequence context. Every base of the mt genome is interrogated by hybridization of fluorescent-labeled DNA fragments against 8 different 25-mer probes forming two probe quartets, one quartet for the light (forward) and one for the heavy (reverse) mtDNA strand. These probes vary only at their central nucleotide position (grey bars with letter in the middle) to account for the 4 possible alleles A, T, C, G or combinations thereof. The 12 bases up-and downstream of the variable position match the rCRS [10]. They constitute the local sequence background for hybridization. Polymorphisms within the flanking nps compromise hybridization. Due to the high mutation rate, multiple polymorphic sites within a window of 25 nps are more frequent in the hypervariable than the control and coding regions. To address the problem of compromised hybridization, the MitoChip v2.0 carries additional alternative probes that match not only the rCRS, but also common variants thereof. The MitoChip v2.0 systematically probes for known hybridization context alternatives including insertions, deletions, and closely spaced single nucleotide polymorphisms. These additional probes embed their middle position not into the context of the reference sequence, but into the context of known variants using alternative flanking probe sequences. Figure 2 illustrates the number of different contexts, available for each position that is covered by at least one additional context.

The alternative context probes, however, have been mostly ignored to date in studies employing the MitoChip v2.0 [11-18,20,21]. This has been due to the lack of software for the automated analysis of multiple local context probe data.

Resequencing analysis of mt genomes operates in two modes: In the qualitative mode, a genotype, namely A, C, G, T, AC, AG, AT, CG, CT, GT, ACG, ACT, AGT, CGT, ACGT or N, is called for each nucleotide position (np). In the quantitative mode, allele proportions are determined in case of heteroplasmic nps. The challenge in both modes is to improve the analysis by using alternative local context probe information.

For nuclear DNA, Two software tools, namely the RATools and the Affymetrix GeneChip Sequence Analysis Software (GSEQ), are used to call bases from hybridization signal intensities. Both are based on the ABACUS algorithm [22]. Position by position, ABACUS screens a sequence of fluorescence intensities and calculates likelihoods for each base independently for both the forward and the reverse strand. For haploid data, five possible calls (Null, A, C, G, T) are examined. For diploid data 6 possible diploid calls (AC, AG, AT, CG, CT, GT) are considered in addition. The pixel intensities within a probe are modeled as independent random variables with a common mean and variance. Means and variances are estimated by maximum likelihood. Forward and reverse strand information is combined by adding the respective (log-)likelihoods. A base call is issued if the difference between the two highest log likelihoods exceeds a given quality score threshold (QT). Otherwise the letter 'N' is introduced into the sequence indicating that no conclusive evidence is available for this position.

ABACUS can be run in two modes to infer the genotype: The haploid mode only calls the four bases A, C, G, T or the letter N. It assumes the genotype to be homozygous, respectively in the case of mtDNA homoplasmic. The diploid mode calls all unsorted pairs of bases. Thus it detects both homozygous (homoplasmic) as well as heterozygous (heteroplasmic) genotypes. Note that ABACUS is designed for genotyping nuclear DNA, where heterozygous positions consist of two alleles present at a ratio of 1:1. For mtDNA the situation is different: As a cell may hold up to 100,000 mt genomes, allele proportions at a heteroplasmic site may vary over a wide range and even comprise more than two alleles, albeit rarely. Nevertheless, due to the lack of alternatives the ABACUS algorithm has been established as a quasi-standard for MitoChip base calling [15,17] propose algorithms building on the output of ABACUS for the detection of heteroplasmy in mtDNA ignoring alternative context probes.

Here we focus on using the alternative context probes arrayed onto the MitoChip v2.0. We introduce ReseqChip, a novel, free and open source software implemented in Perl that exploits data from these alternative local mt genome context probes in the automated consolidation of base calls. ReseqChip does not use raw intensities as input but base calls for alternative local background probes. Ideally, intensities are strongest and likelihoods are highest for the correct base with a clear gap to the runner-up. This is typically observed, if the individual sequence matches the reference sequence both to the left and the right of the base in question in a window of 25-bases in total (the size of the oligonucleotide probe). However, local accumulation of polymorphic sites around the base in question compromises hybridization signals.

Mismatches between the individual sequence and the probe sequence on the array are no longer restricted to the middle base but also occur in the flanking regions. Intensities do not only become weaker, they are also often more uniformly distributed across the four bases, resulting in many N-calls. ReseqChip consolidates these calls. It improves both sensitivity and accuracy compared to applying ABACUS alone.

## Implementation

### Algorithm

Input of ReseqChip are base calls from one of the two ABACUS implementations GSEQ or RATools (RA_Basecaller). The program accepts FASTA formated base caller output files for one or a series of chip experiments. Base calls need to be provided for the reference context as well as all additional contexts. In case of multiple local context probes for the same position the basecallers provide separate calls for each context. Our algorithm aims at identifying the best matching context. We do this indirectly by excluding those probes that appear inadequate. Note, that if a local background is inadequate for a certain position, it will also be inadequate for neighboring positions. Hence, an indication of inadequacy is the accumulation of N-calls. We investigate calls in neighborhoods of length *k *around each sequence position and in each available context. Figure 1B gives an example. In addition to calls obtained using the reference sequence context (left most column), base calls obtained from 6 alternative local context probes (Con_1-6) are available. Note that these alternatives can be related to sequence variants resulting not only from single-base substitutions, but also from insertions or deletions. For the latter, gaps need to be introduced to align homologous sequence positions. The row referring to the current sequence position in the re-sequencing run is highlighted in grey. Neighborhoods of size *k *= 3 (not counting gaps) are shown as black letters. For probe filtering, the parameter max*N *is introduced, i.e. the maximum number of N-calls allowed within the neighborhood. All sequence calls exceeding max*N *are then excluded from further analysis. In the example, max*N *was set at 0, hence only probes producing no N-calls were chosen. They are indicated by stars. In case the calls for the grey row are widely identical across the filtered columns, the actual call is based on majority voting. More precisely, we introduce the additional parameters min*P *and min*U*. If more than min*P *probes remain after filtering and more than min*U *percent call the base *x*, where *x *is the most frequently called base, then *x *is included in the final sequence, otherwise the letter N. High values for min*P *ensure robust base calls, while high values for min*U *ensure a strong majority vote. In the example, min*P *was set to 2, hence the 4 probes remaining after filtering are sufficient for base calling. Moreover, min*U *was set to 50% in the example. Since 3 out of 4 probes call the base, ReseqChip detects the insertion. ReseqChip is implemented in Perl and accepts a FASTA formated file that constitutes the base calls for one or a series of chip experiments as the output of the base caller. Base calls have to be provided for the genotypes with respect to the reference context as well as the additional contexts. The optimal parameter settings derived from the parameter training (see section Parameter Tuning) are provided in a separate parameter file (see Additional file 1) that can be used in conjunction with the test script (see section Availability and Requirements). It allows the user to choose a threshold for the maximum number of N-calls per 100 nps or to define an own parameter set.

### Parameter Calibration

ReseqChip operates on a number of parameters that need to be jointly calibrated. We use training data of known genotype (gold standard) and run ReseqChip on it. We judge parameter dependent performance based on:

1. *dn *- the number of discordant base calls between ReseqChip and the gold standard

2. *nc *- the number of N-calls

There is a trade-off between *dn *and *nc*. Small *dc *values lead to high *nc *values and vice versa. We use a grid of values for one measure and minimize the second measure given the former. The user can then specify the maximal number of N-calls and ReseqChip runs with parameters minimizing the number of discordant call given the user specified restraint on the N-calls. Vice versa, the user can set a restraint on *dc *and receives parameters minimizing *nc*. We let the grid values of both *nc *and *dc *vary from 0 to 20 in steps of 0.1.

## Results

### MitoChip v2.0

We have applied ReseqChip to data from the MitoChip (GeneChip^® ^Mitochondrial Resequencing Array 2.0), a 36 kb oligonucleotide array. The array queries 16,544 nps of the mt genome [10] in 25,413 (25,032 if one not distinguish between the order of PM and MM probes (see below)) different, unique contexts, with each context is represented by two probe quartets, one each for the forward and reverse strand, respectively. Each probe quartet consits of one probe that matches the sequence perfectly (PM) and three mismatched probes (MM) for the three alternative alleles. There are 11,529 (11,910) redundant probes that serve as internal controls of hybridization reproducibility and provide 2 to 70 fold redundancy. That is, the array carries 36,942 probe quartets querying both rCRS [10] and common variants [23] thereof (Table 1). The numbers of interrogated variants, regardless of their contexts are summarized in Table 2. These variants cover 233 different nps.

**Table 1:** MitoChip v2.0 (I)

match	# PQs	# unique PQs (A)	# unique PQs (B)
rCRS	18,824	16,544	16,544
non rCRS	18,118	8,869	8,488

Total	36,942	25,413	25,032

Number of probe quartets (PQs), unique PQs discerning (A) and not discerning (B) the probes representing the PM and the three MMs that match the rCRS and variations of them, respectively.

**Table 2:** MitoChip v2.0 (II)

Type of Variation	# unique	# all
Single-base substitution	246	1365
Deletion	28	102
Insertion	20	94

Total	294	1561

Number of variations that are interrogated by the MitoChip v2.0 additional probes.

### DNA Samples and Chip Experiments

123 mt genome sequences were analyzed using conventional dye-terminator sequencing as well as MitoChip v2.0 resequencing. Dye-terminator sequences constituted the gold standard that we aimed to recover using MitoChip v2.0 data. Among these sequences, 112 were sequenced completely and had been reported previously [19,24], while 19 were analyzed only with respect to the HVS I and II. In addition, pseudo-heteroplasmatic samples were generated for 11 mt genomes by mixing DNA at a ratio of 1:1. All pairwise combinations of mixed DNA where generated in two disjoint groups of 5 and 6 individuals resulting in 10 and 15 controlled heteroplasmic samples, respectively. For these individuals gold standard sequences by dye-terminator sequencing using a published protocol [25] were available. Combining this data with the mixing protocols yielded gold standard genotypes for the artificial heteroplasmic sites. In total we analyzed 148 genomes, whereof 25 were artificial mixtures. This data harbored 172 variants at 159 nps, from which 13 were triallelic. Five of the variants were insertions, 3 were deletions. The total numbers of variable sites were 1190, 246 and 15 for substitutions, insertions and deletions, respectively. The artificially created mixtures contained 205 single nucleotide substitution and 14 insertion type heteroplasmies at 41 variable sites. The polymorphic sites as discovered by dye-terminator sequencing are summarized in Table 3. For every genome we generated base calls with GSEQ for Quality Threshold (QT) values of 3,6,9 and 12 using both haploid and diploid settings.

**Table 3:** Dataset (I)

	# Inds	# Pos/Ind	# Pos
Total	148		89,244 (92,204)
Train	49	603 (623)	29,547 (30,527)
Test	99		59,697 (61,677)

Number of nps involved in the training and test data w/o and with (in brackets) insertions.

Relevant for our analysis were only those nps, that were interrogated by at least one alternative local context probe set. There were a total of 733 nps that met this criterion. Twenty additional nps cover insertions. In total 21,121 probe quartets interrogated these 753 positions, whereof 9,602 were unique. On average about 13 different contexts were assigned to each of the 753 position. Since the 125 contiguous nps around the center of Figure 2 were covered by only one additional context and probes only for less than 1.1% of the contexts, we excluded these positions and restricted our further analysis to the remaining 608 (628) nps. We further excluded nps 107-111 of the rCRS owing to persistent wrong hybridization signal, resulting from a repetitive sequence pattern, which reduced the total number of positions to 603 (623). We randomly selected 49 samples to form a training set for calibrating parameters and collected the remaining 99 for evaluating genotyping performance. The total number of positions involved in the training and testing process and the number of variants and variable sites are listed in Tables 3 and 4.

**Table 4:** Dataset (II)

		Sub	Del	Ins	Total
Homoplasmic	All	985 (166)	15 (3)	246 (3)	1246 (172)
	Train	336 (110)	4 (3)	86 (2)	426 (112)
	Test	649 (143)	11 (2)	160 (3)	820 (148)

Heteroplasmic	All	205 (39)	0	14 (2)	219 (41)
	Train	75 (36)	0	6 (2)	81 (38)
	Test	130 (39)	0	8 (2)	138 (41)

Total	All	1190 (166)	15 (3)	260 (3)	1465 (172)
	Train	411 (116)	4 (3)	92 (3)	507 (122)
	Test	779 (143)	11 (2)	168 (3)	958 (148)

Number of variable sites in the dataset are listed and stratified by type of variation and homoplas-mic/heteroplasmic state. (In brackets the number of distinct SNPs are given.)

### Parameter Calibration

We ran ReseqChip on the training data for various parameter constellations. Discrepancies between MitoChip and the gold standard were considered array-based sequencing errors and nps, for which no call had been generated were counted as N-calls. Let *dc *be the number of sequencing errors and *nc *the number of N-calls. The goal was to keep both measures small. ReseqChip builds on the base calls generated by GSEQ, which depend on the Quality Threshold *QT*. Hence *QT *is an additional parameter that needs to be jointly calibrated with the ReseqChip internal parameters. We repeated our analysis using a range of discrete *QT *values (*QT *= 3,6,9,12). Moreover, we varied the four ReseqChip intrinsic parameters, namely the neighborhood size (*k *= 0,1,...,12), the maximal number of N-calls for probe filtering (max*N *= 0,1,...,5), the minimal number of high quality probes (min*P *= 1,...,11), and the uniqueness threshold (min*U *= 30%,...,100%). In total, 27,456 parameter combinations were tested. The challenge was to jointly tune this set of parameters so that acceptable performances were obtained for both, the number of N-calls and sequencing errors.

**Figure 3:**
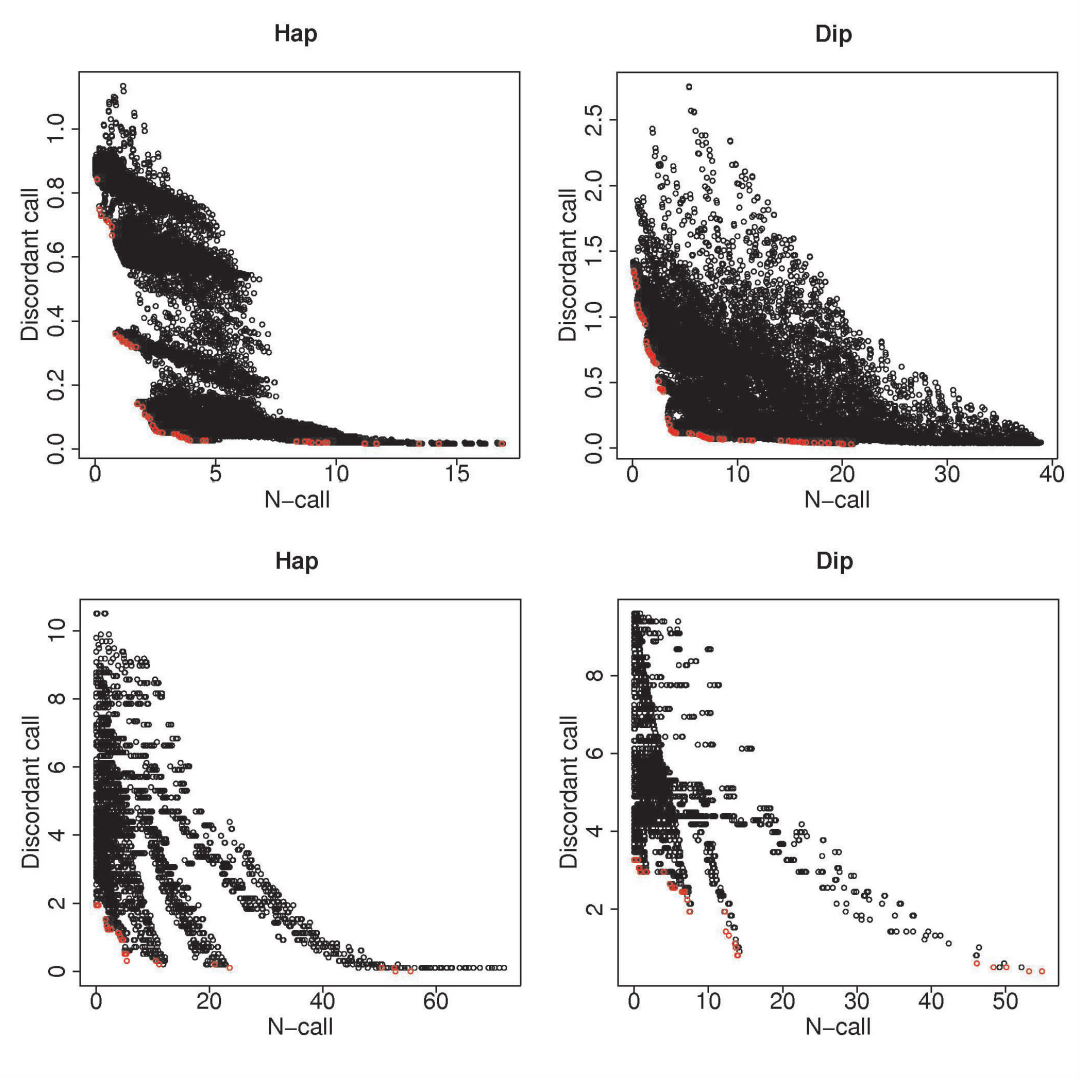
**Performance on Train Data**. The plots show the performance of the 27,456 different parameter constellations in terms of number of sequencing errors and N-calls for substitutions/deletions (top) and insertions (bottom) for haploid (left) and diploid (right) settings of GSEQ.

Figure 3 shows the *nc *and *dc *performance for all 27,456 constellations of parameters assuming a haploid and a diploid model, respectively. Note that there is a trade-off between N-calls and sequencing errors. They cannot be optimized simultaneously. For example, increasing max*N *lead to a smaller number of N-calls, albeit at the expense of more sequencing errors, while lowering min*U*, min*P *and *k *has the opposite effect. Depending on the application, the balance between *nc *and *dc *can be adjusted. ReseqChip allows the user to specify a bound on either *nc *or *dc *and then uses the training data to optimize the parameters with respect to the second error measure. For example, if bounds are set to *nc *we use the data in Figure 3 for calculating the minimum *dc *for increasing *nc*. Optimal settings are indicated by the red dots at the bottom boundary of the cloud. For these points, the x-axis represents the bound to *nc*, while the y-axis gives the optimal *dc *performance achievable under this constraint. The optimal parameter constellations as obtained by the training procedure are listed in Additional file 1.

### Parameter Testing

**Figure 4:**
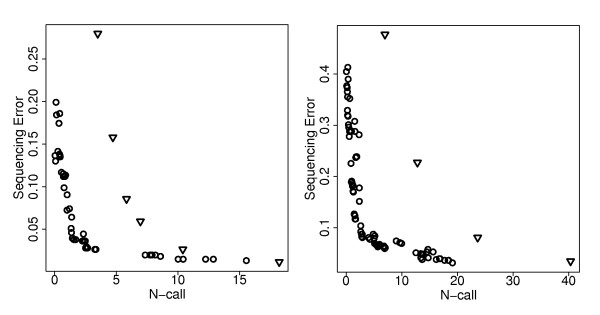
**Performance on Test Data**. The performance of ReseqChip for the test dataset based on the number of sequencing errors and N-calls normalized to 100 nps is plotted for substitutions/deletions with haploid (left) and diploid (right) settings of GSEQ. The circles represent the performance of the optimal parameter constellations obtained from the training. The triangles represent the performance of running GSEQ for QT values of 3,6,9,12,18,24 (but without subsequent sequence assembly by ReseqChip). In the diploid settings only QT values of 3,6,9,12 are shown.

Note that the performances indicated by the red dots in Figure 3 are training set performances. They might be overoptimistic estimates of performance for independent data sets. We evaluated the performance of ReseqChip on independent data using the optimal parameter settings derived from the training data. Figure 4 shows for substitutions/deletions only the results that were obtained applying the various parameter constellations to the test sets for the haploid and the diploid model, respectively. The circles and triangles display the average numbers of discordant (dc) and noncalled (nc) bases normalized to 100 nps that were observed upon inclusion (using ReseqChip) and exclusion, respectively, of the local context probes applying primary base calls only generated by GSEQ for *QT *values of 3, 6, 9, 12, 18 and 24. Evidently, ReseqChip reduces considerably both the number of sequencing errors and the number of N-calls in comparison to a sequence assembly ignoring the alternative local context probes.

## Discussion

ReseqChip allows for the automated consolidation of multiple base calls from alternative local mt genome context probes into a single call, thereby improving the accuracy of mt genome resequencing, while simultaneously reducing the number of N-calls. Based on primary base calls only, it shall be feasible to use ReseqChip with any base calling algorithm and re-sequencing array carrying multiple local context probes. However, this remains to be demonstrated.

Exemplified by the MitoChip v2.0 and the GSEQ basecaller, we demonstrated that an additional sequence consolidation step for analyzing the additional base calls using ReseqChip improved both the accuracy and the call rate. Interestingly, almost all optimal parameter constellations incorporate a *QT *value of 3. This indicates that the algorithm performs best with a low number of N-calls, which in turn gives a reasonable number of possibly conflicting local contexts that can be evaluated. Vice versa, a sequence assembly based on less but more accurate base calls is inferior.

Despite the improvements in call rate and accuracy achieved by means of ReseqChip, it seems impossible to achieve call rates similar to those of conventional sequencing at equal accuracy for at least two reasons: First, array-based resequencing is facing inherent difficulties with respect to variability of the target sequence. There are almost always individual target sequences that do not match some contexts well. Secondly, the principles underlying the hybridization of target sequences to surface-attached probes are still not completely understood. Though discrimination between signal intensities of PM and MM in DNA/DNA hybridization is relatively precise, some PM and MM probes show an unfavorable relation between their signal intensities. This is in particular due to base composition of probe sequence, neighboring bases of PM and MM, and the MM itself [26]. This implies, however, if several probe quartets probing the same or a slightly different local context yield primary base calls that are persistently wrong, then also the consensus call calculated by ReseqChip is wrong. That contributes to the fact that the number of N- and discordant calls cannot become zero.

Our results also show that base calls generated in a diploid model give less accurate calls and more N-calls. For this reason, we have also not pursued allele ratios other than 1:1, as it is obvious that accuracy will decrease with decreasing frequency of the variant allele. We believe that this limitation can be approached only at the level of the basecaller.

## Conclusions

We provide ReseqChip, a freely available software for the automated generation of consensus base calls from resequencing arrays that carry multiple local context probes on the MitoChip v2.0. ReseqChip processes both homozygous (homoplasmic) as well as heterozygous (heteroplasmic) base calls of many chips, hence enabling high-throughput generation of sequence data. ReseqChip significantly improves both accuracy and call rate.

## Availability and Requirements

Project name: ReseqChip

Project home page: The project is part of the live branch of the BioPerl project (bioperl-live): http://code.open-bio.org/svnweb/index.cgi/bioperl/browse/bioperl-live/trunk/Bio/Microarray/Tools/

Availability: the BioPerl live distribution can be downloaded via anonymous svn checkout: svn co svn://code.open-bio.org/bioperl/bioperl-live/trunk bioperl-live

The module path for ReseqChip is Bio/Microarray/Tools. A test script (t/Microarray/Tools/ReseqChip.t) together with test data and optimized parameter set are provided in separate files. The latter files reside in t/data/and carry the prefix "ReseqChip ".

Operating systems: Cross platform

Programming language: Perl

Other requirements: Perl interpreter, BioPerl 1.4 or higher, Statistics-Frequency-0.03 from CPAN http://search.cpan.org/dist/Statistics-Frequency/

License: GNU GPL

## Authors' contributions

Critical revision of the manuscript for important intellectual input: RS and PJO. Technical and material support: HN and WP. Study supervision: PJO. Study concept and design: PJO and RS. Software development: MT. Analysis of data and data interpretation: MT, CL, RS, PJO. Drafting of the manuscript: MT. All authors read and approved the final manuscript.

## Supplementary Material

Additional file 1 **Optimal parameter settings**. A CSV file listing the optimal parameter settings obtained from the training for haploid/diploid model and Single base substitutions-Deletions/Insertions variants. Click here for file
